# Gut Commensal *Bacteroides thetaiotaomicron* Promote Atherothrombosis via Regulating L-Tryptophan Metabolism

**DOI:** 10.31083/j.rcm2511395

**Published:** 2024-11-07

**Authors:** Honghong Liu, Siqin Feng, Muyun Tang, Ran Tian, Shuyang Zhang

**Affiliations:** ^1^Department of Cardiology, Peking Union Medical College Hospital, Chinese Academy of Medical Sciences & Peking Union Medical College, 100730 Beijing, China; ^2^State Key Laboratory of Complex Severe and Rare Diseases, Peking Union Medical College Hospital, Chinese Academy of Medical Sciences & Peking Union Medical College, 100730 Beijing, China

**Keywords:** gut microbiota, coronary artery disease, myocardial infarction, atherothrombosis, *Bacteroides thetaiotaomicron*, L-tryptophan

## Abstract

**Background:**

Coronary thrombosis events continue to be the leading cause of morbidity and mortality worldwide. Recently, emerging evidence has highlighted the role of gut microbiota in cardiovascular disease, but few studies have systematically investigated the gut microbiota variation associated with atherothrombosis.

**Methods:**

We conducted multi-omics analysis (metagenomics sequencing and serum metabolomics) on 146 subjects from Peking Union Medical College Hospital-Coronary Artery Disease (PUMCH-CAD) cohort. We analyzed the key strains and metabolic pathways related to coronary artery disease (CAD) development, explored the bacterial functional pathway which contributes to atherothrombosis at strain level in depth. Single strain colonization procedures on germ free mice demonstrated the promotion of platelet activation and thrombotic phenotypes of the disordered gut microbiota.

**Results:**

Gut microbiome and serum metabolome shifts were apparent in cases of CAD progression, *Bacteroides spp.* disturbed the development of CAD by participating in lipopolysaccharide (LPS), menaquinone and methanogenesis pathways. Particularly, coronary thrombosis is characterized by increased circulatory levels of L-tryptophan, which correlate with *Bacteroides thetaiotaomicron* that has enriched biosynthetic potential. In germ free mice we demonstrate that *Bacteroides thetaiotaomicron* colonization could induce thrombosis, aggravate platelet hyperreactivity and augment fecal levels of L-tryptophan.

**Conclusions:**

The disordered gut microbiota of CAD contributed to the occurrence and development of atherothrombosis. The key members of the bacterial and metabolic features may become biomarkers for predicting the cardiovascular thrombosis event. Targeting the microbial pathway may have the potential to reduce the incidence of cardiovascular disorders.

**Clinical Trial Registration:**

ChiCTR2000033897, https://www.chictr.org.cn/showproj.html?proj=55023.

## 1. Introduction

Coronary artery disease (CAD), a major burden of cardiovascular diseases (CVDs), is a chronic inflammatory pathophysiological process of the artery arising at sites of disturbed blood flow. CAD is attributed to atherosclerosis progression where thrombus formation may cause fatal complications such as myocardial infarction (MI) [1]. Despite timely reperfusion by primary percutaneous coronary intervention, MI is still the main cause of death worldwide [2]. Many researchers have attempted to find novel targets to predict and prevent this critical complication. Since Wang *et al*. [3, 4] reported a metabolite derived from gut microbiota, trimethylamine N-oxide (TMAO), was predictive of CVD events. Gut microbiota has been implicated as a novel ‘endocrine organ’ that plays an important role in CVDs. Thus, further studies are warranted to increase understanding of pathophysiological mechanisms between gut microbiota and CAD, in order to shed light on novel risk biomarkers and interventions.

Many researchers have suggested that commensal microbiota functioned as an environmental factor contributing to metabolic disease [5], atherosclerotic lesions [3, 6] and arterial thrombosis [7, 8, 9]. For instance, Kelly *et al*. [10] analyzed gut microbiota from patients suffering from heart disorders and found *Alloprevotella*, *Prevotella*, and *Paraprevotella* were linked to increasing CVD risk. In another metagenomics study on patients diagnosed with symptomatic atherosclerotic plaques, *Collinsella* was significantly enriched, whereas *Eubacterium *and* Roseburia* were more abundant in healthy volunteers [11]. Our previous study proved that the bacterial co-abundance group (CAG) in the different stages of CAD were dominated by *Roseburia*, *Clostridium IV*, *Klebsiella* and *Ruminococcaceae spp*. [12]. However, the current model cannot effectively differentiate between stable CAD and an MI event, which limits its broad utilization in clinical settings. Therefore, it remains controversial how gut microbiota influences the rupture of atherosclerotic plaque with subsequent thrombus events.

Gut microbiota is responsible for a number of factors related to platelet function and thrombosis, including serotonin [13], vitamin K [14] and von Willebrand factor [15]. Additionally, TMAO can alter calcium signaling in platelets, enhancing platelet reactivity and thrombotic potential [7]. Since gut microbiota are the major source of physiologically pattern-recognition receptors (PRR) agonists, it is crucial to explore the mechanism of thrombogenesis in the microbial ecosystem [16]. Bacterial irritant molecules from microbiota can affect immune vigilance, thereby increasing the risk of thrombosis. By combining metagenomics studies with fecal microbiota transplantation experiments, it can provide relevant microbial-relatedevidence for thrombosis prevention.

To overcome these challenges, we launched interplay analysis of gut microbiome, serum metabolome and clinical indicators in a population of 146 subjects from Peking Union Medical College Hospital CAD cohort (PUMCH-CAD, ChiCTR2000033897, https://www.chictr.org.cn/showproj.html?proj=55023). We characterized multi-omics associations with dominant clinical CAD phenotypes including atherosclerosis burden and thrombotic event type. Especially *Bacteroides *enriched in CAD populations showed a strong correlation with thrombotic events by interfering with L-tryptophan and methanogenesis pathways. Last, we evaluated the ability of microbial signatures including *Bacteroides thetaiotaomicron (B.t)* to modulate platelet function.

## 2. Methods

### 2.1 Cohort Study

#### 2.1.1 Study Design and Subjects

This is a cross-sectional study, where recruited patients were individuals who had been hospitalized for coronary angiography in our center, as previously described [12]. This study was approved by the local Medical Ethics Committee of Peking Union Medical College Hospital (PUMCH) (JS-1195). Prior to their participation in the study, all subjects provided written informed consent. CAD definition: ≥50% stenosis in at least one main coronary artery. Patients were stratified into the following subgroups: (1) MI: defined as a rise of cardiac troponin with at least one value above the 99th percentile upper reference limit and with at least one of the following: (*i*) ischaemia symptoms; (*ii*) new or presumed new significant ST-segment-T wave changes or new left bundle branch block; (*iii*) development of pathological Q waves in the electrocardiogram; (*iv*) imaging evidence of new viable myocardium loss or new regional wall motion abnormality; and (*v*) identification of an intracoronary thrombus by angiography or autopsy [17]; (2) Stable coronary artery disease (SCAD): based on the presence of chest pain that did not change in pattern in the preceding 2 months [18]; and (3) Control: adolescents who exhibited negative results upon coronary computed tomography angiography (CCTA) or coronary angiography examination or were identified as having no CAD-related clinical signs and symptoms. The inclusion and exclusion criteria were previously summarised in accordance with the established criteria [12]. Subjects were excluded if they were diagnosed with gastrointestinal diseases, tumors, autoimmune/infectious diseases, a history of an abdominal operation in the previous year, or had been administered antibiotics for more than three days in the previous three months.

#### 2.1.2 Phenotyping and Metadata Collection

The burden of coronary atherosclerosis was estimated using the Gensini and Syntax scores, as previously reported [12]. Blood pressure was measured with a standard mercury sphygmomanometer when the patient was in a seated position after ≥5 min of rest. Participants’ body mass index (BMI) was calculated as weight (kg) divided by squared height (m^2^). For the MI patients, peripheral blood was drawn from the radial or femoral artery. For the SCAD and controls, peripheral fasting blood was drawn in the morning the day after admission. Peripheral blood samples were centrifuged at 3000 rpm for 5 min and the supernatant was purified. All information provided during the interviews was recorded in the case report form.

### 2.2 Statistical Analysis of Metadata

Continuous, normal distributed variables were expressed as means ± standard deviations. Data which were not normally distributed were described by medians with interquartile ranges. A Student’s *t*-test or non-parametric Mann-Whitney U test was used to analyse data between groups. Categorical data were summarized by numbers and percentages, chi-squared test or Fisher’s exact test were applied when appropriate. All tests were two-sided. *p* values less than 0.05 were considered statistically significant. Statistical analysis was performed using IBM SPSS Statistics version 25 (IBM Inc., Armonk, NY, USA).

### 2.3 Metagenomics Analysis

#### 2.3.1 Stool Sample Collection and DNA Extraction

We provided a stool sampler and detailed instructions for sample collection. Freshly collected stool samples were immediately frozen at –80 °C. DNA extraction was performed using the Qiagen QIAamp DNA Stool Mini Kit (51504, Qiagen, Hilden, Germany) according to the manufacturer’s instructions. DNA quantity was determined using a NanoDrop spectrophotometer, Qubit Fluorometer (with the Quant-iT™dsDNA BR Assay Kit, Q33130, Eugene, OR, USA), and gel electrophoresis.

#### 2.3.2 DNA Library Construction and Sequencing of Fecal Samples

DNA library construction was mainly performed following the manufacturer’s instruction (Illumina, San Diego, CA, USA). We constructed one paired-end (PE) library with an insert size of 350 bp, followed by high-throughput sequencing with PE reads of length 2 × 100 bp. High-quality reads were obtained by filtering low-quality reads with ambiguous “N” bases, adapter contamination, and human DNA contamination from the Illumina raw reads, and by trimming low-quality terminal bases of reads simultaneously [19].

#### 2.3.3 Metagenomic Sequencing and Gene Catalogue Construction

Employing the same parameters to construct the Metagenomics of the Human Intestinal Tract (MetaHIT) gene catalogue [20], we performed gene prediction using SOAPdenovo v1.06 (Beijing Genomics Institute, Hongkong, China) [21] and GeneMark v2.7 (Georgia Institute of Technology, Atlanta, GA, USA) [22]. Genes were aligned using BLAT (BLAST-like alignment tool, Kent Informatics, Inc, Mountain View, CA, USA) and genes with over 90% of their length aligned to another with more than 95% identity were removed as redundancies.

#### 2.3.4 Taxonomic Annotation and Abundance Profiling

We performed taxonomic assignment using an in-house pipeline, we collected the microbial reference genomes from the Integrated Microbial Genomes (IMG) database to make alignment. We used 85% identity as the threshold for genus assignment, and threshold of 80% for the alignment coverage based on sequence similarity across phylogenetic ranks by MetaHIT [23]. For genes, the highest scoring hit(s) above these two thresholds were chosen for the genus assignment.

#### 2.3.5 Diversity Analysis

We calculated α-diversity using the Shannon index based on genus level in order to estimate the microbiome richness. A high α-diversity normally indicates abundant richness of genera within the sample. Principal component analysis (PCA) was analysed on the genus level, and distance-based redundancy analysis (dbRDA) was calculated using Bray–Curtis distance as previously described [24].

#### 2.3.6 Kyoto Encyclopedia of Genes and Genomes (KEGG) Analysis

Differentially enriched KEGG (KEGG database release 59.0, genes from animals and plants removed) pathways were identified according to their reporter score as follows. Wilcoxon rank-sum tests were performed on all KOs of our samples and adjusted for multiple testing using the Benjamin–Hochberg procedure. An absolute reporter score value higher than 1.6 (95% confidence on either tail, according to normal distribution) was used as threshold.

#### 2.3.7 Pathway Functional Characterization

For instance, leucine biosynthesis-related KEGG pathway details were obtained from KEGG database ‘M00432’ (list of KO genes) under the ‘Valine, leucine and isoleucine biosynthesis’ category. Prevalence of genes higher than 5% were analyzed for any of the stages (SCAD, MI) compared to the controls. Pathways shown in our results were manually modified according to KEGG database or referring to the literature. We showed KO genes with significant differences between group comparisons (*p* < 0.05).

### 2.4 Metabolomics Analysis

#### 2.4.1 Untargeted Metabolomics Study and Serum Metabolite Data Analysis

Metabolomics analysis was performed on a Waters ACQUITY ultra-high-performance liquid chromatography system (Waters Corporation, Milford, MA, USA) coupled with a Waters Q-TOF Micromass system (Waters Corporation, Milford, MA, USA) as previously described [25, 26]. We used databases such as KEGG, MetaboAnalyst, Human Metabolome Database, and METLIN to identify metabolic pathways. Next, SIMCA-P 14.0 software (Umetrics AB, Umea, Sweden) was implied to acquire metabolite information and variables.

#### 2.4.2 Clustering of Co-Abundant Serum Metabolites

A total of 7061 serum metabolic features were yielded after pre-processing above. We conducted a “cross-comparison scheme” as follows to downsize metabolic features associated with disease status. (*i*) Multi-comparisons set A: various stages of CAD were compared with healthy control (HC) and to each other using the Wilcoxon test and *T* test, the metabolites will be retained for the next stage of analysis both statistics results satisfied the adjusted *p* value (q value) < 0.05 meanwhile the fold change >1.2 or <0.83; (*ii*) Multi-comparisons set B: compare the metabolites between the four groups and HC vs. CAD, metabolites were reserved when any test of Jonckheere-Tespstra test or Kruskal. test satisfied *p* value < 0.05. We then integrated the two collections, serum metabolomics profiling yielded 562 features after removing the drug metabolites.

We cluster metabolites into metabotypes using the R package WGCNA [27] as previously described. For parameters, scale-free topology threshold was set for β = 14 and deepSplit threshold was set for 4 [28]. The serum metabolites clusters (labelled M01–M35) were collectively termed serum metabotypes.

#### 2.4.3 Targeted Serum Metabolomics Analysis

All standards were obtained from Sigma-Aldrich (St. Louis, MO, USA), Steraloids Inc. (Newport, RI, USA) and TRC Chemicals (Toronto, ON, Canada). All the standards were accurately weighed and prepared to obtain individual solutions at a concentration of 5.0 mg/mL. Formic acid was of analytical grade obtained from Sigma-Aldrich (St. Louis, MO, USA). Methanol (Optima LC-MS, Thermo Fisher Scientific, Waltham, MA, USA), acetonitrile (Optima LC-MS), and isopropanol (Optima LC-MS, Thermo Fisher Scientific, Waltham, MA, USA) were purchased from Thermo-Fisher Scientific (FairLawn, NJ, USA). Sample preparation was performed as previously described [26]. Targeted quantitation was performed to determine the concentration of metabolites as previously described by reverse-phase UHPLC using a Prominence 20 UFLCXR system (Shimadzu, Columbia, MD, USA) with a Waters (Milford, MA, USA) BEH C18 column (2.1 × 100 mm × 1.7 µm particle size).

### 2.5 Spearman Multi-Omics Correlation Analysis

Spearman correlations between functional modules, serum metabolites, and clinical parameters were calculated using R (version 4.3.0), and both differential abundances of AS- or Thrombosis-associated functional modules and metabolites were tested using the Wilcoxon rank sum test. Wherever mentioned, the Benjamini-Hochberg method was used to control the false discovery rate (FDR).

### 2.6 Animal Study

#### 2.6.1 Mice and Diets

Germ-free (GF) *ApoE* -/- mice were provided by the Institute of Laboratory Animal Sciences (ILAS) at the Chinese Academy of Medical Sciences and Peking Union Medical College [research license no. SYXK (Beijing) 2015-0035], which is a member of (and accredited by) the American Association for the Accreditation of Laboratory Animal Care. All experiments were performed in accordance with the guidelines of the Institutional Animal Care and Use Committees of the ILAS. The mice were maintained under standard GF conditions with a 12:12-hour light:dark cycle. GF mice were maintained in flexible film isolators and their GF status was checked weekly by aerobic and anaerobic culture. All GF *ApoE* -/- mice were male. All mice were used after reaching the age of 6 weeks. All GF *ApoE* -/- mice received sterilized high fat diet (40 kcal% fat and 1.25% w/w cholesterol; product number D12108S) which was purchased from Changzhou SYSE Bio-Tec. Co., Ltd (Changzhou, Jiangsu, China).

#### 2.6.2 Bacteria Preparation and Gavage Study

*Bacteroides thetaiotaomicron* (ATCC 29741) and *Prevotella copri* (control strain, ATCC 33547) were cultured in tryptic soy agar/broth supplemented with defibrinated sheep blood (ATCC medium 260) under anaerobic conditions. Strains were harvested in logarithmic phase, aliquoted using fresh broth and concentrated to 10^9^ CFU/mL and diluted into 10% glycerol under anaerobic conditions and stored at –80 °C until use. GF *ApoE-/-* mice were kept in individual isolators and received a high-fat diet. In the next three weeks, the mice were gavaged twice a week, each time with 100 µL of bacterial solution containing *B. thetaiotaomicron* or *P. copri* (5 × 10^8^ CFU/mouse).

#### 2.6.3 In Vivo Carotid Artery Thrombosis Models

Measurement of acute thrombus formation were performed as described earlier [29]. Some mice did not survive meaning there were ultimately 8 mice in the *B.t *group and 7 mice in the *Prevotella copri (P.c)* group. After the mice were anesthetized, the left jugular vein and right carotid artery were carefully dissected. The mice were injected intravenously with Rhodamin B to label the platelets. The ferric chloride injury model was performed by placing 7.5% FeCl_3_ solution for 1 min laterally to the common carotid artery. Then, the resulting thrombus formation and occlusion of the artery was recorded using a high-speed wide-field Leica M205 FCA fluorescence stereo microscope with Leica Application Suite X (LAS X) software (Leica Microsystems GmbH, Wetzlar, Germany). We recorded the occulusion time. The experiment was terminated if the carotid artery did not occlude within 30 minutes.

#### 2.6.4 Platelet Isolation and Platelet Suspension Preparation

Mouse platelet-rich plasma (PRP) was prepared as described previously with minor modifications [30, 31, 32]. Mouse whole blood (≈900 µL) was taken by orbital and collected into a sodium citrate blood collection tube and gently shaken upside down 8 times. PRP (≈600–700 µL) was separated by centrifuging at 200 g for 15 min. The pellet was resuspended in 500 µL HEPES Tyrode’s buffer (pH 7.4) and centrifugation was repeated (to fully remove other blood cells) at 200 g for 5 min (room temperature), then the supernatant was aspirated.

#### 2.6.5 In Vitro Platelet Activation and Flow Cytometry Assay

The final platelet suspensions (100 µL PRP dilute to 700 µL with HEPES Tyrode’s buffer (pH 7.4); 2 × 10^7^ platelets/mL) were recovered for 30 min at room temperature prior to the experiments. Washed platelets for experiments were used within 4 h after isolation. Agonists of collagen were added separately at different concentrations (10 µg/mL, 15 µg/mL and 20 µg/mL) and a blank was kept as a control. The platelets were incubated with agonists for 10 min at room temperature. APC-conjugated anti-P-selectin (CD62P, 148304, Biolegend, San Diego, CA, USA) was added to each tube and incubated in the dark for 15 min. The platelet suspensions were then fixed with 100 µL of 4% paraformaldehyde. Data was acquired on a flow cytometer (Accuri™ C6 plus, BD Biosciences). Twenty thousand (20,000) events were acquired. The data was analyzed using FlowJo v10.4 software (BD Biosciences, San Jose, CA, USA).

#### 2.6.6 Platelets Adhesion under Flow

For the platelet spreading functional study, we largely followed procedures as previously described [31]. Microfluidic shear flow experiments were performed using the rectangular flow chamber assay (31-010, Glycotech, Rockville, MD, USA) equipped with single channel syringe pump NE-1000 and flexcell VP750 vacuum pump. Briefly, cover glasses were degreased (24 by 60 mm) by incubating overnight in undiluted chromosulfuric acid. Then the glass coverslips were coated with collagen (100 µg/mL; C7661-10MG, Sigma, St. Louis, MO, USA) and fibrinogen (100 µg/mL; F3879, Sigma). The coated coverslips were blocked with Tyrode’s HEPES buffer containing 1% bovine serum albumin (BSA). The rested and washed platelets were stained by FITC labeled anti-CD41Mab (1:100; 133903, Biolegend) in the dark at room temperature for 30 min. Unbound antibodies were washed away and prostacyclin was added at a final concentration of 10 ng/mL to prevent platelet activation. The platelet pellet was reconstituted in HEPES Tyrode buffer to achieve a platelet concentration of 150,000 platelets/µL. After the incubation, platelet samples were perfused over chips coated with or without agonist at a physiological shear rate (7.5 µL/min) using a microfluidic device for 20 min. Immediately after performing the flow experiments, samples were fixed for 15 minutes in 4% paraformaldehyde in PBS at room temperature in the dark. Microscopic images were acquired using a Nikon (Tokyo, Japan) confocal microscope with a 60× oil immersion objective. Image analysis was performed using ImageJ software (1.53a, Wayne Rasband, USA) predominantly as described previously [33]. In short, 15 consecutive images along the length of the channel and covering the proximal, middle, and distal regions of the flow channel were acquired, converted into 8-bit grayscale images, and underwent adjustment for brightness and contrast. Images of stained platelets were quantified using ImageJ software. Intensity threshold was chosen to select for specific staining and quantified for integrated optical density.

## 3. Results

### 3.1 General Characteristics of the Current Cohort

To explore the role of gut microbiota in mediating cardiac events, we conducted a prospective cohort study of patients with CAD at PUMCH, China. In total, 36 control subjects and 110 CAD patients who underwent coronary arteriography were included, CAD patients were divided into a SCAD subgroup (N = 64) and an MI subgroup who were suffering from a coronary thrombosis event (MI, N = 46). The inclusion and exclusion criteria were previously summarized in methods. The MI subgroup showed a more severe atherosclerosis burden than the SCAD group in terms of the Gensini score and the Syntax score (evaluate severity of plaque burden under coronary angiography). The number of stenosed vessels was also significantly increased (Gensini score, *p* < 0.001, SCAD vs. MI; Syntax score, *p* < 0.001, SCAD vs. MI). Besides, myocardial necrosis including cardiac troponin I (cTnI, *p* = 0.021, SCAD vs. MI) and creatine kinase-muscle/brain (CK-MB, *p* = 0.011, HC vs. MI) were markedly elevated in the MI group (Table 1). Traditional CVD risk factors including age, smoking status, systolic blood pressure, fasting blood glucose and estimated glomerular filtration rate (eGFR), exhibited no difference between the SCAD vs. MI groups. We observed that SCAD patients showed lower levels of low-density lipoprotein cholesterol (LDL-C) than MI patients (*p* = 0.008) which may be attributed to a higher usage ratio of statins. In conclusion, the current cohort showed that MI patients suffered from higher atherosclerosis severity and thrombosis burden compared to the SCAD patients.

**Table 1.** **Characteristics of the current cohort**.

Group		HC (N = 36)	SCAD (N = 64)	MI (N = 46)	*p* value
Gender (Male, %)^§^		22 (61.1%)	51 (79.7%)	36 (78.3%)	0.097^a^
Age, years*		58.24 ± 10.7	60.86 ± 9.5	61.28 ± 10.7	0.177
SBP, mmHg*		125 ± 17.3	129.8 ± 13.7	128.9 ± 20	0.161
BMI, kg/m^2^*		24.6 ± 3.8	26.6 ± 3.2	26.1 ± 3.4	0.005^a⁢b^
Current smoker^§^		14 (38.9%)	39 (60.9%)	26 (56.5%)	0.097^a^
Gensini score^†^		NA	40.8 (33.1, 57.8)	74.5 (56.6, 93)	<0.001^c^
Syntax score^†^		NA	9 (5.3, 13)	17 (10.8, 23.1)	<0.001^c^
No. of stenosed vessels^§^					0.089
	1	NA	18 (38.1%)	1 (2.2%)	
	2	NA	21 (32.8%)	10 (21.7%)	
	3	NA	25 (39%)	35 (76.1%)	
NYHA class^§^					0.13
	1	36 (100%)	43 (67.2%)	33 (71.7%)	
	2	0	18 (28.1%)	12 (26.1%)	
	3	0	3 (4.7%)	1 (2.2%)	
TIMI risk score		6.8 (3.5, 9.9)	9.9 (6.8, 14.5)	9.9 (6.8, 14.5)	<0.001^a⁢b^
Laboratory data					
	WBC×10^9^/L*	6.47 ± 1.9	7.07 ± 2.2	7.13 ± 2.19	0.209^b^
	ALT, U/L^†^	19.5 (15, 28.5)	23.5 (19.3, 33.8)	27.5 (20, 37)	0.004^a⁢b^
	LDL-C, mmol/L^†^	2.65 (1.9, 3.3)	1.9 (1.6, 2.5)	2.3 (1.9, 2.9)	<0.001^a⁢c^
	Hs-CRP, mg/mL^†^	1.03 (0.42, 2.3)	1.3 (0.5, 2.6)	2.7 (1.1, 6.3)	0.001^b⁢c^
	FBG, mmol/L^†^	6 (5.2, 7.1)	6.2 (5.3, 8.2)	7 (5.9, 7.9)	0.059^b^
	CK-MB, µ/L^†^	0.5 (0.4, 0.85)	0.7 (0.5, 1)	0.75 (0.5, 1.3)	0.028^a⁢b^
	cTnI, µg/L^†^	0	0.00 (0, 0.01)	0.28 (0.08, 0.78)	0.015^a⁢c^
	Cr, umol/L^†^	73.5 (66, 85.3)	79 (69.3, 89.5)	81.5 (70, 91)	0.033^a⁢b^
	eGFR, mL/min/1.73 m^2⁣†^	94.7 (83.2, 111.7)	91.7 (82.7, 105.2)	89.2 (81.6, 111.9)	0.533
	PCI history^§^	0	45 (70.3%)	36 (56.5%)	<0.001^a⁢b^
	Stroke^§^	0	2 (3.1%)	6 (13%)	0.020^a^
	Diabetes mellitus^§^	5 (13.9%)	18 (28.1%)	17 (37%)	0.066^b^
	HTN^§^	7 (19.4%)	41 (64%)	34 (73.9%)	<0.001^a⁢b^
	PAD^§^	1 (2.8%)	20 (31.3%)	13 (28.3%)	0.003^a⁢b^
Drugs					
	Aspirin^§^	0	60 (93.8%)	46 (100%)	<0.001^a⁢b^
	Clopidogrel^§^	0	49 (76.6%)	38 (82.6%)	<0.001^a⁢b^
	β-blocker^§^	4 (11.1%)	49 (76.6%)	40 (87%)	<0.001^a⁢b^
	ACEI/ARB^§^	5 (13.9%)	43 (67.2%)	23 (50%)	<0.001^a⁢b^
	Statin^§^	1 (2.8%)	60 (93.8%)	40 (87%)	<0.001^a⁢b^
	Metformin^§^	2 (5.6%)	11 (17.2%)	7 (15.2%)	0.251
	PPI^§^	2 (5.6%)	10 (15.6%)	7 (15.2%)	0.309

^†^median (IQR), *mean ± SD, ^§^n (%). Continuous and distributed variables were analyzed by one-way analysis of variance. The Kruskale Wallis H-test was applied for data which were not normally distributed. Student’s *t*-test were used to analyse continuous, normally distributed data. Mann-Whitney U test was applied for data of this type that were not normally distributed. Categorical variables were compared by the χ^2^ test. ^a^*p* < 0.05 for Control and SCAD comparison. ^b^*p* < 0.05 for Control and MI comparison. ^c^*p* < 0.05 for SCAD and MI comparison. Abbreviation: SBP, systolic blood pressure; BMI, body mass index; NYHA, New York Heart Association; WBC, white blood cell; ALT, alanine aminotransferase; LDL-C, low-density lipoprotein cholesterol; Hs-CRP, high-sensitivity C-reactive protein; FBG, fasting blood glucose; CK-MB, creatine kinase-muscle/brain; cTnI, cardiac troponin I; Cr, creatinine; eGFR, estimated glomerular filtration rate; PCI, percutaneous coronary intervention; HTN, hypertension; PAD, peripheral artery disease; ACEI, angiotensin-converting enzyme inhibitor; ARB, angiotensin II receptor blocker; PPI, proton pump inhibitor; SCAD, stable coronary artery disease; MI, myocardial infarction; HC, healthy control; TIMI, thrombolysis in myocardial infarction; NA, not applicable; IQR, interquartile range.

### 3.2 Changes in the Gut Microbiome between MI and SCAD Patients

**Fig. 1.**
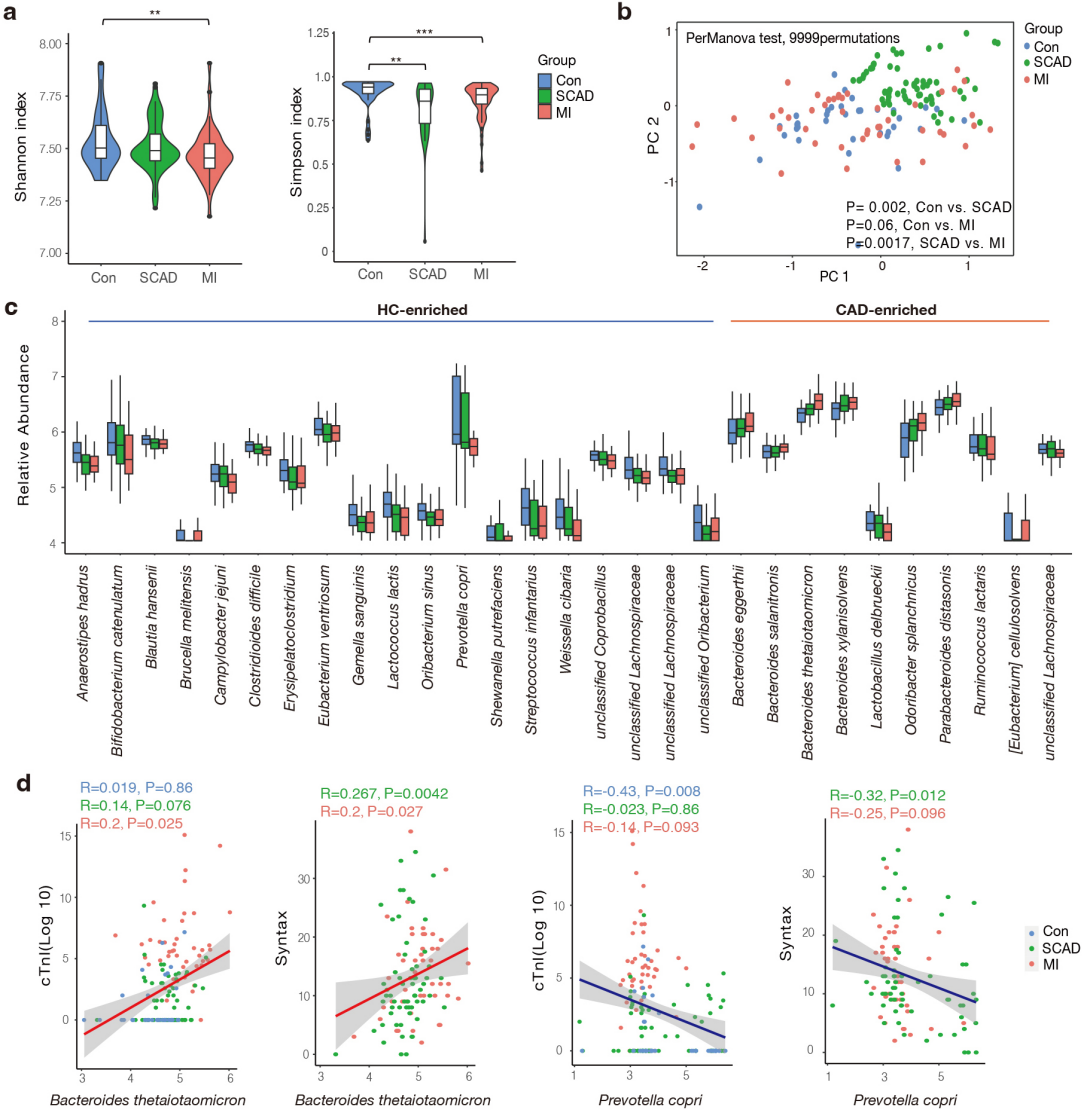
**Gut microbial characteristics of the cohort**. (a) SCAD and MI patients were characterized by lower microbial richness in Shannon and Simpson indexes based on genera level compared to healthy controls. (b) Differentially changed bacteria feature in SCAD, MI and Control according to distance-based redundancy analysis based on the Bray-Curtis distance. perMANOVA test, 9999 permutations, *p* values were annotated. (c) Species significantly changed in the CAD compared to Control group (*p* value < 0.01 and fold change >1.2 or <0.83). (d) Spearman correlation between significantly altered species and disease severity indicator. Wilcoxon rank sum test; **, *p* < 0.01; ***, *p* < 0.001. Con, control subjects; SCAD, stable coronary artery disease; MI, myocardial Infarction; CAD, coronary artery disease; HC, healthy control; PC, principal component.

A total of 146 fecal samples were subjected to metagenomic shotgun sequencing in order to investigate the variations in the gut microbiome. Following the removal of low-quality reads and human DNA reads, 52.4 million high-quality reads were aligned to a comprehensive gene catalogue, resulting in an average mapping rate of 87.2 ± 1.6% for each sample (**Supplementary Table 1**). The microbiome data in this study are available at (https://db.cngb.org/cnsa/), project number: CNP0001804. CAD patients showed much lower Shannon and Simpson indices compared to the control group at the genus level (Fig. 1a). We constructed dbRDA score plot based on Bray-Curtis distances to depict the overall structure of gut microbiota. The composition of the microbiota signature differed significantly even in the SCAD vs. MI comparison (Fig. 1b).

Then, we aimed to screen dominant bacteria with prognostic value for CAD progression (**Supplementary Table 2**). In total, 19 species were elevated in the control group while 10 species were more abundant in the CAD group, respectively (Fig. 1c). These CAD-enriched bacteria, including *Bacteroides thetaiotaomicron*, *Bacteroides xylanisolvens* were positively correlated to AS burden and atherothrombotic risk represented by the thrombolysis in myocardial infarction (TIMI) risk score (Fig. 1d and **Supplementary Fig. 1**) [34]. In accordance with the findings of previous studies, members of the *Lachnospiraceae* family are the primary producers of short-chain fatty acids, showed dominant relevance in healthy subjects [35]. We found *Prevotella copri* was more abundant in the control group, which was negatively associated with Syntax score and cTnI levels (Fig. 1d). It has been reported that *prevotella copri *was more common in the gut microbes who prefer plant-rich diet consumption and mediate glucose response [36]. In summary, we found gut microbiota composition and structure differed between the SCAD and MI groups, and closely correlated with the phenotype of CAD severity.

### 3.3 Integrating Analysis of Microbial Pathways and Metabotypes Associated to CAD Progression

**Fig. 2.**
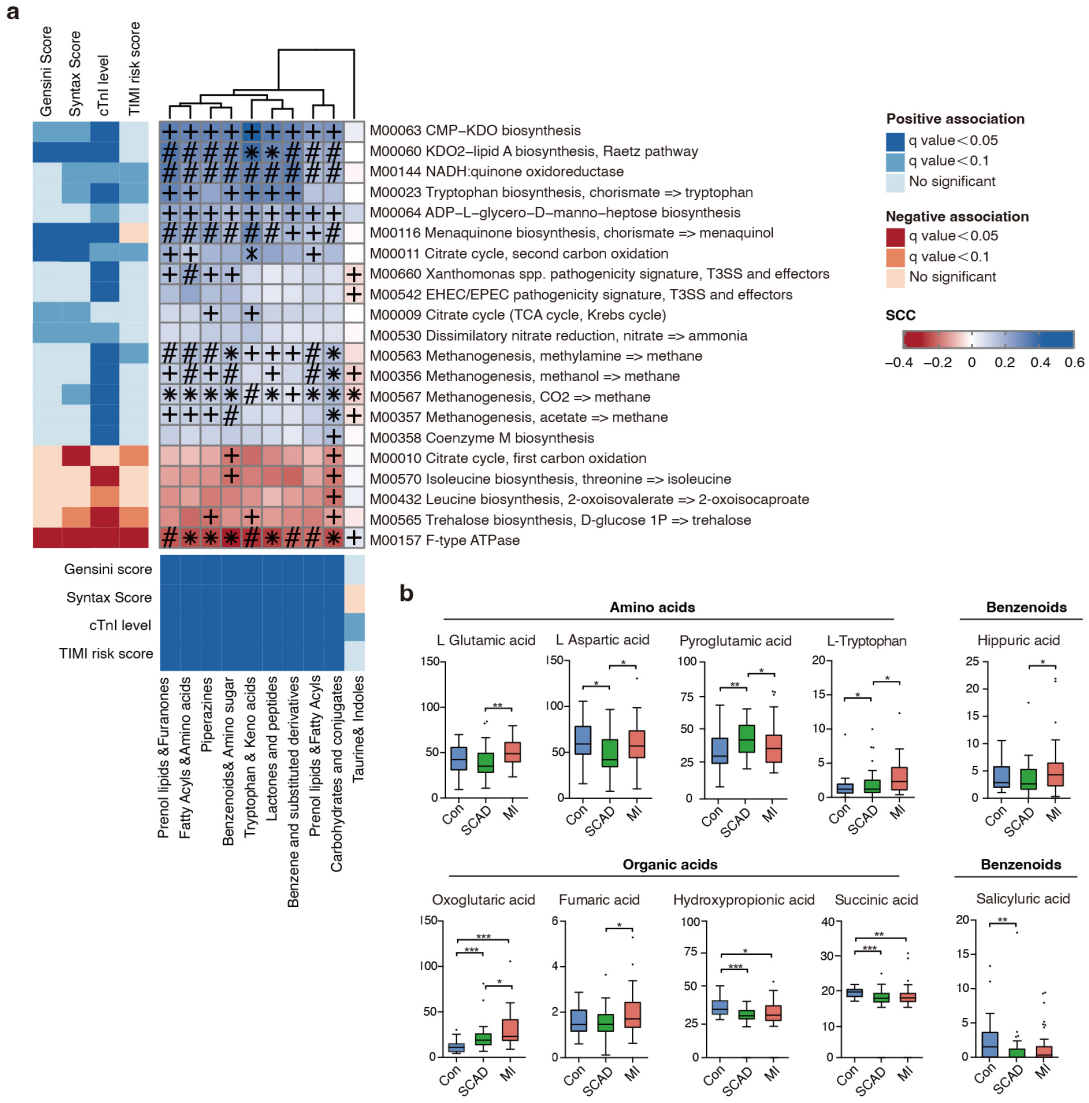
**Fine-grained correlation profile of metagenomics, metabonomics and clinical traits in 146 subjects**. (a) The left and under panel showed associations (Mann-Whitney U-test; FDR <0.1) between microbial KEGG modules or serum metabotypes and clinical phenotypes; different colors indicate a different kind of association. The right panel show corresponding relationships between the gut KEGG modules and serum metabotypes. Coloring represents Spearman correlation coefficient, where FDRs are denoted, +FDR <0.05, #0.05 < FDR < 0.001, *FDR <0.001. (b) Significantly altered serum metabolites in Control and CAD subgroups. Wilcoxon rank sum test; *, *p* < 0.05; **, *p* < 0.01; ***, *p* < 0.001. FDR, false discovery rate; Con, control subjects; SCAD, stable coronary artery disease; MI, myocardial Infarction; CAD, coronary artery disease; KEGG, Kyoto Encyclopedia of Genes and Genomes; KDO2, 2-keto-3-deoxyoctulosonic acid; NADH, nicotinamide adenine dinucleotide (reduced); EHEC, enterohaemorrhagic escherichia coli; EPEC, enteropathogenic escherichia coli; T3SS, type three secretion system; ATPASE, adenosine triphosphatase; ADP, adenosine diphosphate; CMP-KDO, cytidine 5’-monophosphate-2-keto-3-deoxyoctulosonic acid; TCA, tricarboxylic acid cycle (also known as the citric acid cycle or Krebs cycle); cTnI, cardiac troponin I; TIMI, thrombolysis in myocardial infarction; SCC, staphylococcal cassette chromosome.

Since gut microbiota interacts with their host through circulatory metabolic exchange, we integrated gut microbial functions and serum metabolomics in the states of CAD progression. The metabolic potential of gut microbiota was represented by KEGG modules, which are manually curated pathway units consisted of KOs. 21 KEGG modules were remarkably associated with one or more of the CAD phenotypes (**Supplementary Table 3**). Next, we examined 146 serum samples under polar ionic/lipid mode for untargeted metabolomic analysis, and 562 metabolites were further binned into 35 co-abundance metabotypes (**Supplementary Table 4** and **Supplementary Fig. 2**). In accordance with previous reports [37], lipid metabotypes enriched in the control subjects mainly included phosphatidylcholine (PC), phosphatidylethanolamine (PE) and phosphatidylserine (PS). Meanwhile, metabotypes elevated in different stages of CAD included amino acids, prenol lipids, benzenoids, fatty acyls and furanones (**Supplementary Fig. 2**). Importantly, these bacterial KEGG modules were also correlated to CAD phenotype filtered metabotypes (Fig. 2a), with the majority also differing in abundance in the expected direction in the cohort. The bacterial pathways positively associated with CAD progression contained enzymes for lipopolysaccharides (LPS), menaquinone (Vitamin K2) biosynthesis, citrate cycle and various transport systems, which have been proven closely linked to CVD [38, 39]. In contrast, the microbiome negatively associated with CAD progression contained bacterial genes important for leucine and trehalose biosynthesis. Notably, methanogenesis and tryptophan pathway showed a positive correlation with cTnI levels which is an indicator of atherothrombosis. Research has shown methane may exert cardioprotective effects via its anti-oxidative and anti-inflammatory activities [40]. As an essential aromatic amino acid, tryptophan is the precursor of indole derivatives which normally under the direct regulation of the gut microbiota [41].

Since amino acids, bile acids, benzenoids and elements of organic acids were focuses of attention. We next measured 132 serum metabolites using targeted metabolomics in 10 categories and 10 compounds were finally identified as being significantly associated with CAD progression (Fig. 2b, **Supplementary Table 5**). We noticed that L-tryptophan increased with CAD severity in the serum, reflecting a CVD predictable prognosis value. Overall, we investigated the microbial modules in relation to serum metabolites and CAD phenotypes using cross-domain associations, indicating gut microbiota regulate cardiometabolic disease as a novel endocrine organ.

### 3.4 CAD Progression-Associated Changes in Microbial Genes Summarized in KEGG Pathways and Bacteria

**Fig. 3.**
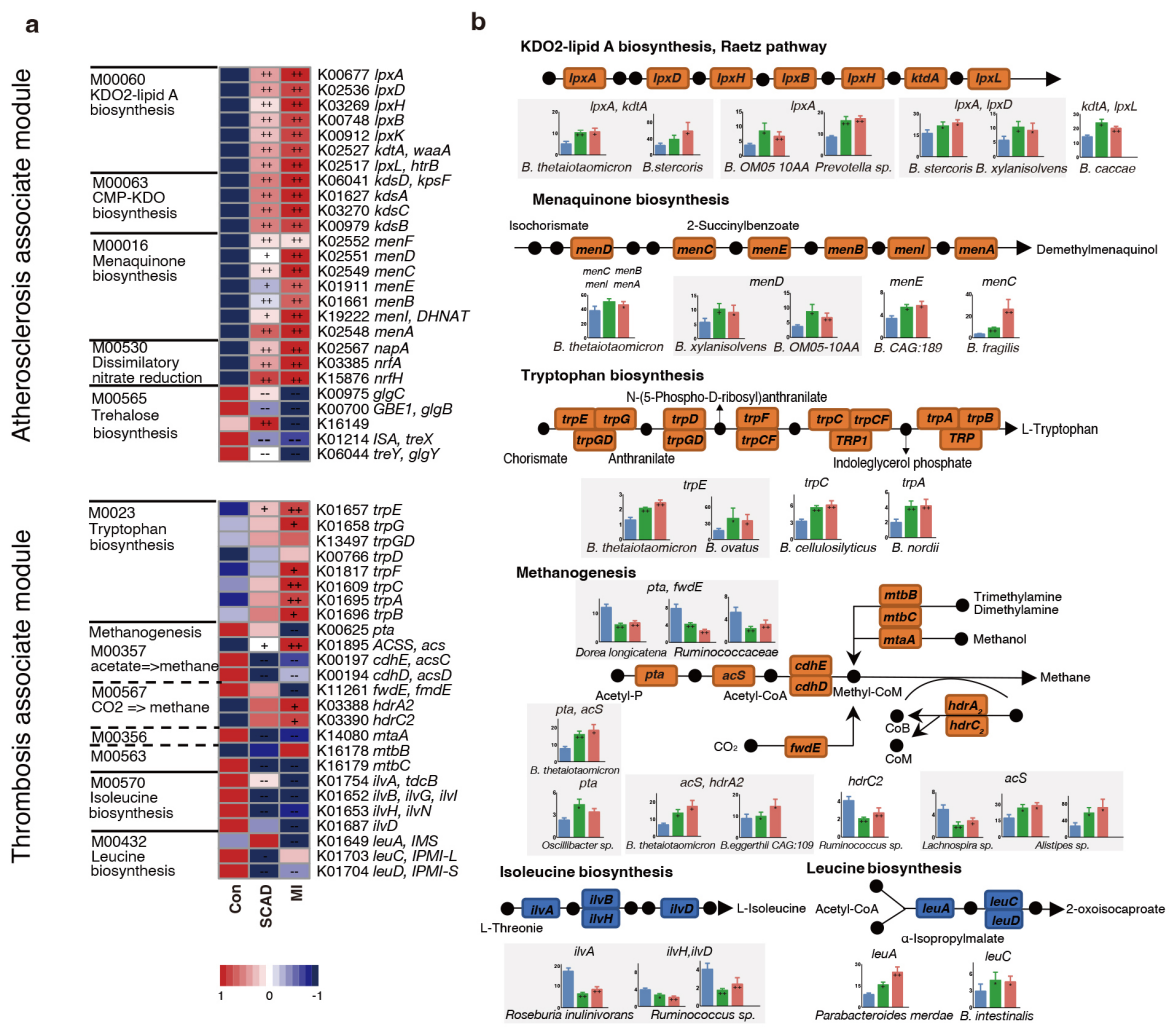
**CAD progression-associated changes in bacteria summarized in KO genes and KEGG pathways**. (a) Relative abundance of KOs altered in each of CAD stages compared to the Control were shown in the heat map. Significant elevation or depletion were denoted as follows: ++, elevation with *p* < 0.01; +, elevation with *p* < 0.05; –, depletion with *p* < 0.01; -, depletion at *p* < 0.05. (b) Representative KO genes were summarized in modified KEGG pathway maps. Microorganisms with the most abundant expression of representative genes were further denoted. Gene abundances within each of subgroups were shown in bar plots. Wilcoxon rank sum test; *, *p* < 0.05; +, *p* < 0.01; ++, *p* < 0.001. Con, control subjects; SCAD, stable coronary artery disease; MI, myocardial Infarction; CAD, coronary artery disease.

In order to check the roles of microorganisms in metabolic pathways, we dissected the critical KOs related to CAD progression and identified the key bacteria which were responsible for KO variation. Among the 5 main atherosclerosis-associated microbial modules, a significant elevation was observed in 21 KOs and a significant depletion in 5 KOs in at least one of the disease stages when compared to the control (Fig. 3a). The alterations in the gene were illustrated in a pathway representation, which was constructed manually by modifying KEGG pathway maps (Fig. 3b). Bacteria expressed lipopolysaccharide (LPS) biosynthesis enzyme A (*lpxA*) and 2-Keto-3-deoxyoctonate transferase A (*kdtA*) gene in 2-keto-3-deoxyoctulosonic acid (KDO2)-lipid A biosynthesis including* Bacteroides thetaiotaomicron*, *Bacteroides stercoris *and *Bacteroides xylanisolvens*, were also found significantly elevated in CAD patients. *Bacteroides *were also strongly related to menaquinone biosynthesis pathway, *Bacteroides sp. OM05-10AA*, *Bacteroides fragilis *and *Bacteroides thetaiotaomicron* were identified as top marker expressing gene menaquinone biosynthesis enzyme C (*menC*) and menaquinone biosynthesis enzyme D (*menD*). *Ruminococcaceae* and *Faecalibacterium prausnitzii* in control subjects showed a positive relationship with AS-negative modules such as trehalose biosynthesis. *Bacteroidetes* may promote thrombosis since they were involved in the synthesis of L-tryptophan for expressing abundant tryptophan biosynthesis gene E (*trpE*), tryptophan biosynthesis gene C (*trpC*) and tryptophan biosynthesis gene A (*trpA*). Overall, *Bacteroidetes *seem to affect the progression of CAD at different stages by participating in LPS, menaquinone and methanogenesis pathways.

In the pathways related to myocardial damage, most of the methane-producing KOs decrease in CAD such as phosphotransacetylase (*pta*) and forward transferase E (*fwdE*). These KOs were negatively correlated with *Ruminococcaceae spp.* while positively correlated with *Bacteroidetes *and *Alistipes sp.* Meanwhile, genes (*ilvA*, *leuA*) involved in the biosynthesis of leucine and isoleucine were significantly depleted in SCAD and MI. *Roseburia inulinivorans* was found to be active in iso-leucine production, while *Parabacteroides merdae* and *Bacteroides intestinalis *reduced amino acid generation. In conclusion, *Bacteroides spp*. were found to exhibit a dominant functional role in CAD progression through metagenomic analysis.

### 3.5 Transmission of B. thetaiotaomicron Provoke Platelet Hyperreactivity and Thrombosis Risk

**Fig. 4.**
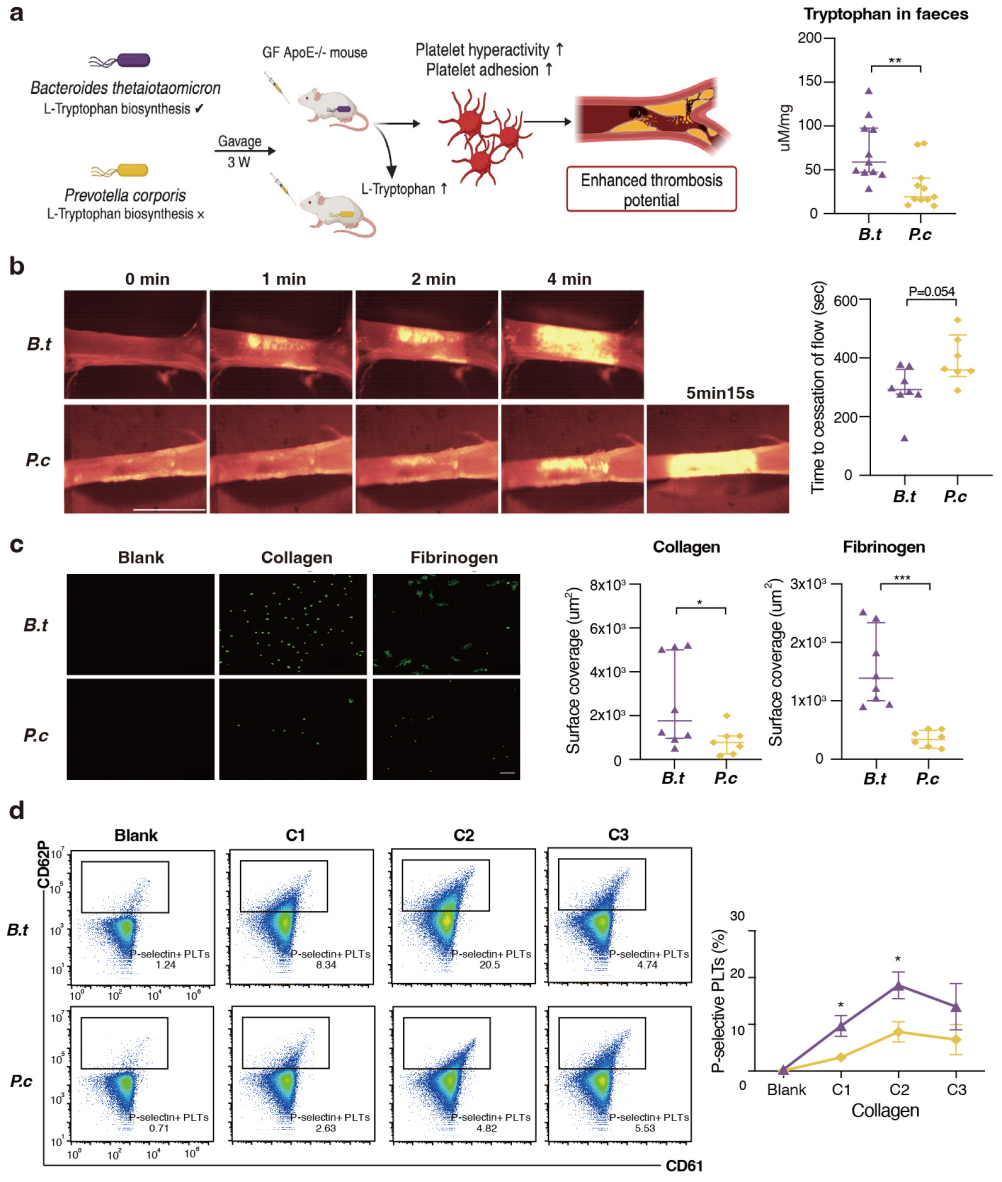
***B. thetaiotaomicron* induce L-tryptophan biosynthesis and provoke thrombosis by microbiota transplantation in germ free mice**. (a) We colonized *Bacteroides thetaiotaomicron* and *Prevotella copri* with germ free *ApoE-/-* mice fed on high-fat diet for three weeks (n = 11 pergroup). L-tryptophan levels in mice faeces gavaged with *B. thetaiotaomicron *or *p. copri *(11 per group). (b) Intravital epifluorescence video microscopy of thrombus formation at different time (representative images) in the FeCl_3_ -injured carotid artery of germ free *ApoE-/-* mice gavaged with *B. thetaiotaomicron *(n = 8) or *p. copri *(n = 7) with analysis of occlusion times. Scale bar 2 mm. (c) Standardized blood flow chamber for platelet adhesion on collagen and fibrinogen. End-stage representative images of platelet coverage area were shown. Scale bar 10 µm. (d) Expressions of platelet activation markers P-selectin (CD62P) *in vitro* by increasing collagen concentrations (10 µg/mL, 15 µg/mL, 20 µg/mL). Data were presented as median with interquartile range except for flow cytometric results. For (a–c), *p* values were determined by either Mann-Whitney U-test; for (d), data were examined by two-way ANOVA. *, *p* < 0.05; **, *p* < 0.01; ***, *p* < 0.001. GF, germ free; *B.t*, *Bacteroides thetaiotaomicron*; *P.c*, *Prevotella copri.*; GF ApoE−/− mouse, germ-free apolipoprotein E knockout mouse; CD62P, P-selectin; CD61, integrin β3.

Since* B. thetaiotaomicron* was the strongest driver strain for L-tryptophan biosynthesis, suggesting a possible causal relationship. Meanwhile, we have observed a robust association between increasing *B. thetaiotaomicron *and major adverse cardiac events in our prospective cohort (Data not published), served as impetus for research aimed at verifying the hypothesis that *B. thetaiotaomicron* may modulate platelet activity. We chose another LPS-producing Gram-negative bacterium *Prevotella copri *as a control strain since this strain does not contain genes encoding *Trp* biosynthesis. Both *Bacteroides* and *Prevotella* have been identified as dominant contributors to human gut enterotypes [23]. To experimentally address the issue, we colonized *B. thetaiotaomicron* and *P. copri* with germ free *ApoE-/-* mice fed on high-fat diet for three weeks. The concentration of fecal L-tryptophan levels in *B. thetaiotaomicron* mice was observed to be approximately twice that of the concentration observed in the *P. copri* mice (Fig. 4a). Notably, mice kept with *B. thetaiotaomicron* showed enhanced *in vivo* thrombosis potential, as we observed that time to blood flow cessation was shortened (indicating pro-thrombotic phenotype) compared to *P. copri *(286.1 ± 33.4 s vs. 391.8 ± 35.6 s, Fig. 4b) by carotid artery following FeCl_3_ injury. Next, we examined the effect of *B. thetaiotaomicron* on platelet adhesion [42]. Washed platelets were fluorescently labeled, and the adherence to the agonist surface under physiological shear forces was monitored. *B. thetaiotaomicron *colonization promotes adhesion-induced platelet activation within anticoagulated whole blood to immobilized collagen and fibrinogen, represented by visible density in fluorescent platelet adhesion (Fig. 4c). Induction of platelet surface activation marker P-selectin (CD62P) is a method for verifying the functional properties of platelets [43]. We observed that platelet activation is induced by collagen in mice colonized with *B. thetaiotaomicron*, leading to a thrombi formation phenotype (Fig. 4d). Cumulatively, the above data demonstrate that *B. thetaiotaomicron *colonization may facilitates adhesion-induced platelet activation and enhance *in vivo* thrombosis potential.

## 4. Discussion

Research recently has revealed an unexpected interaction between gut microbial metabolism and the host to modify the risk of developing CVD [44, 45]. We strengthened the novelty to dissect clinical CAD stage–specific microbial features associated with disease progression by integrating multi-omics for clinical phenotype, gut microbiome, and fasting serum metabolome. The current study directly demonstrated that CAD-associated functional components of gut microbiome: notably the upregulated potential for lipopolysaccharide, L-tryptophan and methanogenesis pathways. Further, *B. thetaiotaomicron* colonization studies confirm that gut commensal may modulate platelet hyperresponsiveness and thrombosis potential. These conclusions contribute to evaluating the relationship between adverse cardiovascular outcomes and imbalanced microbial homeostasis.

Atherosclerotic plaque rupture and thrombosis can lead to severe ischemic events, result in MI and death [46]. Our results demonstrate that microbiome shifted with the progression of CAD compared to controls and highlighted the remarkable role of *Bacteroidetes* in the onset and development of CAD. Members of *Bacteroides spp.* are potential commensals and mutualists of the human colon [47], and they only function as “providers” for microbes residing close to them, but also profoundly affect the susceptibility of the host to inflammatory diseases [48, 49]. While few other studies reported a negative association between *Bacteroidetes* abundance and CVD populations [50, 51, 52], the inconsistency may be due to single case-control comparison analysis design and the CAD patients recruited in these researches contained various subtypes. Meanwhile, CAD patients exhibited higher carbohydrate intake according to our previous study [12], which shed light on the opinion that diet habit may causally select for gut microbial features. As recent research reinforced that gut microbiota modulates the interplay between diet and cardiometabolic disease risk [53]. Based on the current conclusions, the structure in bacteria between different stages of CAD might represent distinct metabolic roles for atherosclerotic plaque growth at early stages and plaque rupture or intra-arterial thrombus at late stages of CAD.

The human metabolome consists of endogenous metabolites and bacteria-derived metabolites. The metabolic patterns observed in patients with varying stages of CAD indicate that atherothrombosis may be associated with a universal metabolic disturbance. Similar to our previous findings, integrating metagenomics and metabolome profiles showed that benzenoids and menaquinols, which are normally biosynthesized by bacteria, significantly perturbed with the development of CAD [12]. The observed associations suggest that *Bacteroides thetaiotaomicron* involved in the synthesis of tryptophan may reflect CVD prognosis linked to MI, potentially play a unique role in the maintenance of platelet function and artery thrombosis. L-tryptophan and corresponding catabolites have been widely studied as key regulators of cardiovascular homeostasis, as well as immune homeostasis [41, 54, 55]. Several cohorts [56, 57] mainly consisting of individuals from Western countries found circulatory tryptophan was inversely associated with cardiac mortality and thrombotic event while downstream indoles metabolites were positively correlated to major adverse cardiac event. We speculate that the inconsistency is attributed to diet difference, and have conducted work using a verification cohort including more than 2000 patients and analyzed the important role of diet.

More emerging evidence has confirmed the relationship between bacteria and thrombosis formation [7, 58, 59]. Gut microbiota, an actuating trigger of a systematic immune response, could affect atherothrombosis and subsequent adverse prognosis. Our data revealed severe clot formation in the *B. thetaiotaomicron* colonization GF mice compared with their *P. copri* counterparts, indicating a stimulatory effect of gut microbe on atherothrombosis. We analyzed platelet hyperresponsiveness on platelet activating matrices to mimic physiological arterial flow conditions, in order to detect differences in platelet activation. Similar to our *in vivo* results, we found *B. thetaiotaomicron* to promote prothrombotic platelet adhesion function. More importantly, vascular inflammation not only “fuels” atherosclerosis but also creates the milieu for episodes of thromboses [60]. In conclusion, given the pivotal role of thrombi in immunothrombosis, it is plausible that the mutualistic relationship between gut microbiota and host not only impairs immunovigilance but also influences arterial thrombus formation under steady-state conditions.

The present study had several limitations that should be considered when interpreting the results. Despite the numerous findings of *B. thetaiotaomicron* on thrombosis potential, the mechanistic insights for bacteria-derived tryptophan within platelets function remains unknown. Although we selected *P. copri* as control strain in order to minimize the interference of LPS, other immune factors may be involved in the formation of thrombi and further research is needed. The current research mainly focused on the association of *B. thetaiotaomicron* with platelet function, and we have also evidenced that long-term colonization of gut microbiota cause formation of atherosclerotic plaques in another study (Data unpublished).

The gut microbial ecosystem is capable of producing a variety of metabolites that are carried via circulation and influence host biological processes by distributing to distant body sites. The microbiome in the human gut may survive, decline or flourish in response to endoenvironmental perturbations. Multi-omic studies could provide a global understanding of bacterial variations that occur in CVD populations as a consequence. Collectively, our findings have implications on future studies that aim to depict microbiome-health association network across disease stages and may enable the design of non-invasive diagnostic tools.

## 5. Conclusions

Since CAD is a chronic, long-term pathologic disease that is closely associated with inflammatory cascades [61]. The underlying mechanism responsible for the sudden transformation of a stable atherosclerotic plaque to thrombosis may cause life-threatening death, which normally occurs after decades of progression [62]. Therefore, seeking novel and effective biomarkers for monitoring plaque rupture is important for prevention of cardiovascular death. Our results show that gut microbiota is closely correlated to CAD progression via the mediation of circulatory metabolites. Furthermore, the present study highlights that coronary thrombosis may be influenced by the tryptophan output of the* Bacteroides spp.*, as well as the presence of microbial-related methanogenesis pathways. The current study provides ideas for understanding the interaction of host-intestinal microbiota in the pathogenesis of atherothrombosis, and modulating gut microbiota as a therapeutic target.

## Availability of Data and Materials

The microbiome data in this study are available at (https://db.cngb.org/cnsa/), project number: CNP0001804.

## Supplementary Material

Supplementary material associated with this article can be found, in the online version, at https://doi.org/10.31083/j.rcm2511395.
