# Supplementary material for: Gut Commensal Bacteroides thetaiotaomicron Promote Atherothrombosis via Regulating L-Tryptophan Metabolism
Source: Rev Cardiovasc Med. 2024 Nov 7;25(11):395. doi: 10.31083/j.rcm2511395 (PMC11607515; doi:10.31083/j.rcm2511395)
Supplement: Supplementary file 1 [file 2153-8174-25-11-395-s1.zip › Supplementary figures.docx]

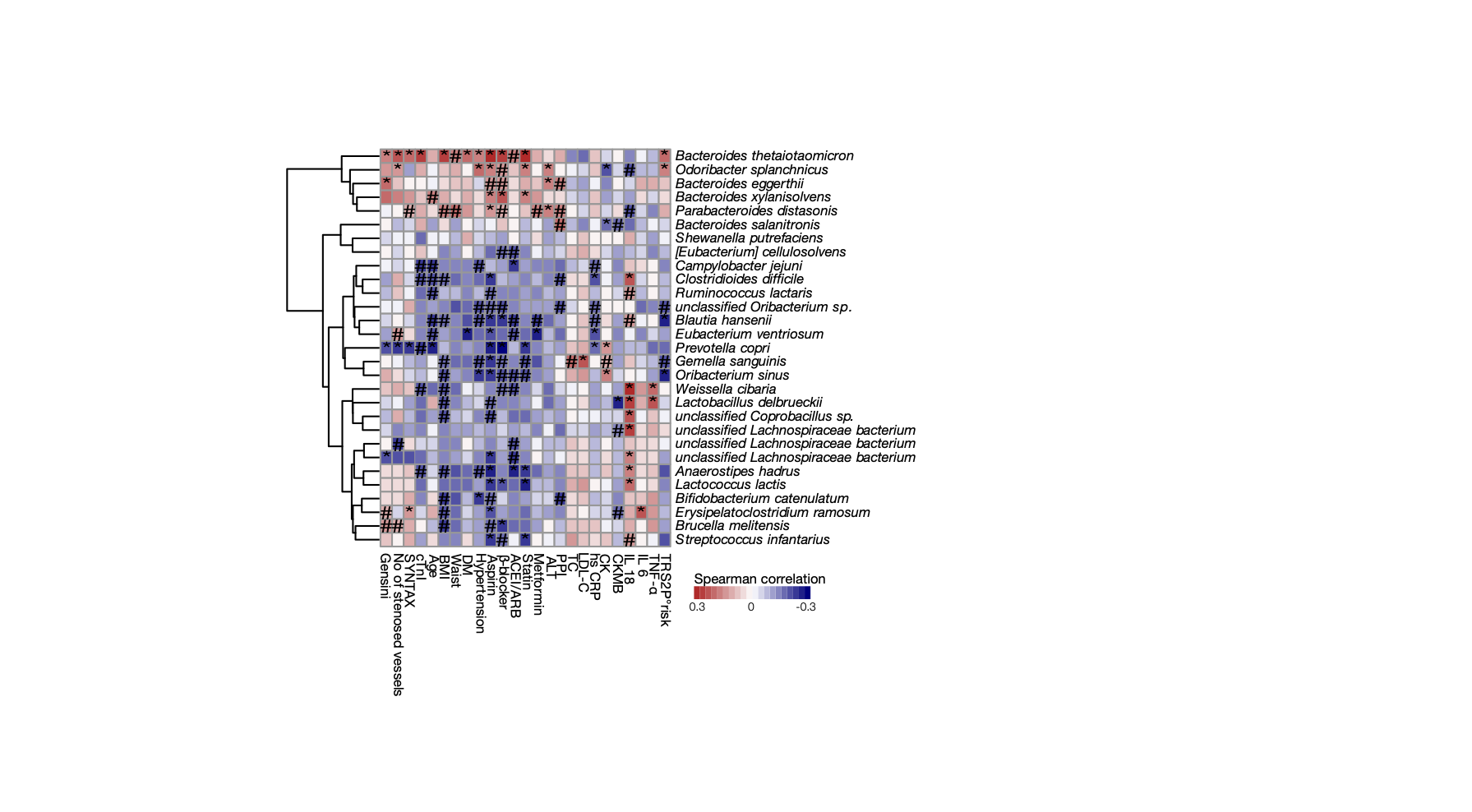


**Supplementary Fig. 1. Associations between varied species and coronary artery disease clinical indicators.** The color represents positive (red) or negative (blue) correlation, and FDRs are denoted as follows: ^#^, FDR < 0.1; *, FDR < 0.05. Abbreviation: BMI, body mass index; DM, diabetes mellitus; ACEI, angiotensin-converting enzyme inhibitor; ARB, angiotensin II receptor blocker; ALT, alanine aminotransferase; PPI, proton pump inhibitor; LDL-C, low-density lipoprotein cholesterol; Hs-CRP, high-sensitivity C-reactive protein; CK-MB, creatine kinase-muscle/brain; cTnI, cardiac troponin I; IL-6, interleukin 6; TNF-α, tumour necrosis factor-α; FDR, false discovery rate.


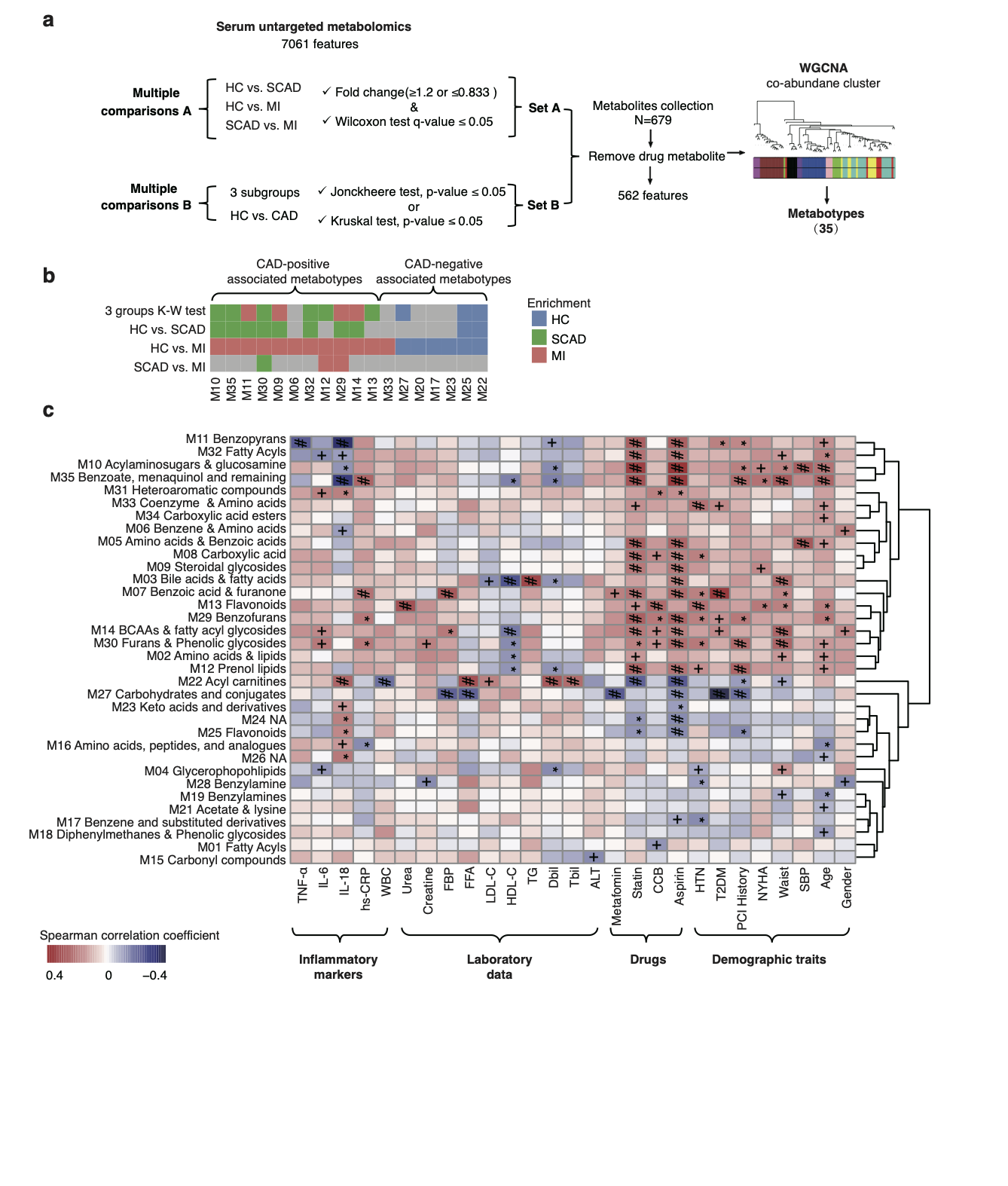


**Supplementary Fig. 2. Profile of fasting serum metabotypes and physiological traits in 146 subjects.** (a) We conducted a “cross-comparison scheme” to identify the serum metabolome features in different CAD groups. Serum metabolites were summarized as ‘metabotypes’, disturbed features were filtered for significant positive or negative associations with CAD phenotype. (b) The serum metabotypes significantly changed between different CAD groups according to the corresponding test. (c) Spearman correlations between all fasting serum metabotypes and clinical phenotypes. The color represents positive (red) or negative (blue) correlations and FDRs are denoted: ^+^, FDR < 0.1; *, FDR < 0.05; ^#^, FDR < 0.01. Abbreviation: FDR, false discovery rate; HC, healthy control subjects; SCAD, stable coronary artery disease; MI, myocardial Infarction; CAD, coronary artery disease; IL-6, interleukin 6; TNF-α, tumour necrosis factor-α; Hs-CRP, high-sensitivity C-reactive protein; WBC, white blood cell; FBG, fasting blood glucose; FFA, free fatty acids; LDL-C, low-density lipoprotein cholesterol; HDL-C, high-density lipoprotein cholesterol; ALT, alanine aminotransferase; HTN, hypertension; CCB, calcium channel blocker; DM, diabetes mellitus; NYHA, New York Heart Association; SBP, systolic blood pressure; PCI, percutaneous coronary intervention.
